# Risk factors for extraurothelial recurrence in upper tract urothelial carcinoma after radical nephroureterectomy: a retrospective study based on a Chinese population

**DOI:** 10.3389/fonc.2023.1164464

**Published:** 2023-08-09

**Authors:** Zhenkai Luo, Binbin Jiao, Yangxuanyu Yan, Caixia Su, Yijin Pan, Hang Zhao, Yuxuan Bo, Guan Zhang, Zhenshan Ding

**Affiliations:** ^1^ Department of Colorectal Surgery, National Cancer Center/National Clinical Research Center for Cancer/Cancer Hospital, Chinese Academy of Medical Sciences and Peking Union Medical College, Beijing, China; ^2^ Graduate School of Peking Union Medical College and Chinese Academy of Medical Sciences, Beijing, China; ^3^ Department of Urology, Beijing Chao-Yang Hospital, Capital Medical University, Beijing, China; ^4^ Peking University, China-Japan Friendship School Clinical Medicine, Beijing, China; ^5^ Department of Urology, China-Japan Friendship Hospital, Beijing, China; ^6^ School of Public Health, Peking University, Beijing, China; ^7^ Department of Head and Neck Surgery, Fudan University Shanghai Cancer Center, Shanghai, China; ^8^ Department of Oncology, Shanghai Medical College, Fudan University, Shanghai, China

**Keywords:** extraurothelial recurrence, upper tract urothelial carcinoma, radical nephroureterectomy, risk factor, Ki-67

## Abstract

**Objectives:**

The risk factors for extraurothelial recurrence (EUR) after radical nephroureterectomy (RNU) in patients with upper urinary tract urothelial carcinoma (UTUC) are currently inconsistent and unclear. In this study, we aimed to identify these risk factors and develop a grading system for EUR.

**Methods:**

We retrospectively analyzed 220 patients who underwent RNU for UTUC in our center from January 2009 to December 2020. Overall survival (OS) and extraurothelial recurrence-free survival (EURFS) were compared using the Kaplan–Meier curve with a log-rank test. Univariate and multivariate Cox regression models were applied to identify the independent risk factors related to EUR.

**Results:**

The median follow-up period was 42 (range: 2–143) months. Of the 220 patients, 61 patients developed EUR in our cohort, which had worse survival outcome. Multivariate Cox regression analysis showed pathologic stage, lymph node (LN) status, lymphovascular invasion (LVI), Ki-67, neutrophil-to-lymphocyte ratio (NLR), and platelet-to-lymphocyte ratio (PLR) were independent risk factors for EUR. The Kaplan–Meier curves revealed a significant difference in EUR among the three risk groups.

**Conclusion:**

Our study suggests that pathologic stage, LN status, LVI, Ki-67, NLR, and PLR are independent risk factors for EUR in UTUC patients after RNU. The development of a grading system for EUR risk stratification may assist urologists in making clinical decisions regarding the management of UTUC.

## Introduction

Upper urinary tract urothelial carcinoma (UTUC) is a relatively rare malignant tumor of the urinary system. Its incidence rate is far lower than that of bladder cancer (BC), accounting for 5%–10% of all urothelial carcinomas (1, 2). The symptoms in the early stage of UTUC are relatively obscure, and the degree of malignancy is relatively high. Approximately 60% of the patients have UTUC that already infiltrated the muscular layer at diagnosis (3, 4). Compared with other urological tumors, the clinical progress of UTUC is faster and the prognosis is worse. Radical nephroureterectomy (RNU) with bladder sleeve resection is the standard surgical treatment of UTUC, especially for patients with high risk (5).

It is noteworthy that patients who underwent RNU still have a high rate of intravesical recurrence (IVR) and extraurothelial recurrence (EUR), leading to poor prognosis (1). A large multi-institution study of 1,363 patients treated with RNU demonstrated that 379 (28%) patients experienced EUR and the median time to EUR was 10.4 months (6). Up to now, most present studies still pay attention to the survival time or IVR of patients with UTUC after receiving RNU. Previous studies reported that pathologic tumor stage, tumor grade, lymph node (LN) status, and lymphovascular invasion (LVI) are independent risk factors for overall survival (OS) after RNU (7, 8). Some blood inflammation biomarkers including neutrophil-to-lymphocyte ratio (NLR), platelet-to-lymphocyte ratio (PLR), and lymphocyte-to-monocyte ratio (LMR) have also been found to be related to survival time (9, 10). However, few studies focused on the risk factors predicting the EUR.

Because EUR plays a crucial role in survival outcome, it is important to identify predictors of EUR to enable early detection and intervention to improve prognosis. In this study, we aimed to identify the risk factors of EUR in UTUC patients undergoing RNU. Additionally, we developed a grading system based on these factors to stratify risk and assist urologists in optimizing clinical decision-making.

## Methods

### Patient selection

This retrospective study was approved by the China-Japan Friendship Hospital Research Ethics Board (2021-40-K24). Informed consent was obtained from all eligible participants in advance. This work has been reported in line with the Strengthening The Reporting Of Cohort Studies in Surgery (STROCSS) guidelines (11). We retrospectively collected the information of patients diagnosed with UTUC who received RNU treatment at our hospital from 2009 to 2020; all patient details have been de-identified. The patients who met the following criteria were included: 1) patients with UTUC confirmed pathologically; 2) patients with primary disease; 3) patients with unilateral onset; and 4) patients subject to RNU combined with cystic sleeve resection. Patients were excluded according to the following criteria: 1) patients with bilateral UTUC; 2) patients subject to no RNU combined with cystectomy; 3) patients with metastatic uroepithelial carcinoma or concurrent BC; and 4) patients with another pathology. None of the patients included in this study received neoadjuvant chemotherapy.

### Data collection

The following clinicopathological features of patients were collected: sex, age at the first diagnosis, body mass index (BMI), tumor laterality, pathologic tumor stage (pT), LN status (pN0, pNx, or pN+), tumor grade, tumor multifocality, LVI, surgical margin status, distant metastasis, and related laboratory examination parameters. Previous history of hypertension and diabetes mellitus (DM), preoperative urine cytology, presence of hydronephrosis on the affected side, and adjuvant chemotherapy were also included. All resected tumor specimens were sent for pathological examination by senior pathologists. The 2009 International Union Against Cancer (UICC)/American Joint Committee on Cancer (AJCC) TNM classification system (12) and the 2016 World Health Organization (WHO) grading system (13) were performed to evaluate the pathologic tumor stage and grade. The estimated glomerular filtration rate (eGFR) was calculated using the equation GFR mL/min/1.73 m^2 = ^194 * (0.739 if female) * serum creatinine^-1.094^ * age^-0.287^. Patients with an eGFR lower than 60 mL/min/1.73 m^2^ were considered to have chronic kidney disease. NLR, PLR, and LMR were obtained from routine blood examination performed 1 week before surgery. We utilized the X-tile software (version 3.6.1) to determine the optimal cutoff for these biomarkers, which were classified as low and high level (14). The cutoff values of NLR, PLR, and LMR were 2.4, 132.6, and 3.2, respectively.

### Follow-up

We monitored patients every 3 months during the first year after surgery, every 6 months through the third year, and once a year thereafter. Follow-up data included blood tests, cystoscopic examination, urinary system ultrasound, chest and abdomen CT, urine exfoliated cytology, and urography. Selective bone scan, PET/CT, or magnetic resonance imaging (MRI) examination was performed if clinically indicated. IVR and recurrence in the contralateral upper urinary tract were not considered EUR in this study. OS was defined as the time from the date of RNU to death from any cause. Cancer-specific survival (CSS) was defined as the time from the date of RNU to the date of cancer-specific mortality. Extraurothelial recurrence-free survival (EURFS) was defined as the time from the date of RNU to the date of the first EUR according to the imaging examination.

### Statistical analysis

We expressed the categorical variables as the frequency (percentage). Continuous variables were presented as median values with ranges. Kruskal–Wallis H test, chi-square test, and Fisher’s exact test were used to analyze variables. Kaplan–Meier survival curves were plotted, and the log-rank test was conducted to demonstrate differences in different risk groups. In addition, the receiver operating characteristic (ROC) curve analysis was performed, and the area under the ROC curve (AUC) was calculated to evaluate the discrimination ability. Prognostic risk factors related to EUR were assessed by univariate and multivariate Cox regression analysis models, and the results were shown as hazard ratio (HR) with 95% confidence interval (CI). All p values were obtained from two-sided tests, and *p* < 0.05 indicated that the difference was statistically significant. R software (Version 4.1.2) and IBM SPSS Statistics (Version 24) were utilized to complete all statistical analyses and figures.

## Results

### Patient characteristics

After screening from the inclusion and exclusion criteria, the data of a total of 220 patients were collected. All patients underwent laparoscopic nephroureterectomy and extravesical incision on bladder cuff management. Some patients underwent mitomycin C (MMC) bladder instillation immediately or within 24 h after RNU. The clinicopathological features of all patients were summarized in **Table 1**. There were 98 men and 122 women. The median follow-up after surgery was 42 months (range, 2–143 months). The median age of all of the patients was 68 years. Of the 220 patients, 61 (27.7%) had EUR; the lung was the most common site of recurrence, followed by the liver, distant LN, bone, and brain. In our study, 58 patients developed IVR. No significant differences were observed in terms of age, sex, BMI, history of hypertension, history of DM, tumor location, tumor laterality, tumor size, tumor multifocality, hydronephrosis, margin positivity, urine cytology, eGFR, and adjuvant chemotherapy among the subgroups of EUR (all *p* > 0.05). Patients with EUR had higher rates of a higher pathologic tumor stage (pT3–4), high-grade tumor, positive LN, LVI, high expression of Ki-67, and inflammation biomarkers (all *p* < 0.05).

**Table 1:** Characteristics of the 220 patients stratified by extraurothelial recurrence.

Characteristic	With EUR	Without EUR	*p* value
	N=61	N=159	
**Age (years)**	66 (40-85)	69 (38-86)	0.295
**Sex**			0.712
Men	32 (52.5%)	66 (41.5%)	
Women	29 (47.5%)	93 (58.5%)	
**BMI**	24.77 (16.44-40.00)	24.22 (16.42-33.25)	0.634
**History of hypertension**			0.828
Yes	29 (47.5%)	80 (50.3%)	
No	32 (52.5%)	79 (49.7%)	
**History of DM**			0.845
Yes	12 (19.7%)	35 (22.0%)	
No	49 (80.3%)	124 (78.0%)	
**Tumor location**			0.800
Ureter	33 (54.1%)	77 (48.4%)	
Renal pelvis	25 (41.0%)	73 (45.9%)	
Both	3 (4.9%)	9 (5.7%)	
**Laterality**			0.651
Left	31 (50.8%)	88 (55.3%)	
Right	30 (49.2%)	71 (44.7%)	
**Pathologic stage**			0.003
pT2 or less	37 (60.7%)	129 (81.1%)	
pT3 or greater	24 (39.3%)	30 (18.9.1%)	
**Lymph node status**			0.050
pN0/pNx	54 (88.5%)	153 (96.2%)	
pN+	7 (11.5%)	6 (3.8%)	
**Tumor grade**			0.044
Low	2 (3.3%)	20 (12.6%)	
High	59 (96.7%)	139 (87.4%)	
**Tumor size**			0.054
<3cm	31 (50.8%)	105 (66.0%)	
≥3cm	30 (49.2%)	54 (34.0%)	
**Tumor multifocality**			1.000
Present	14 (23.0%)	35 (22.0%)	
Absent	47 (77.0%)	124 (78.0%)	
**Hydronephrosis**			0.073
Present	32 (52.5%)	106 (66.7%)	
Absent	29 (47.5%)	53 (33.3%)	
**LVI**			0.046
Present	45 (73.8%)	138 (86.8%)	
Absent	16 (26.2%)	21 (13.2%)	
**Margin positivity**			0.145
Negative	56 (91.8%)	154 (96.9%)	
Positive	5 (8.2%)	5 (3.1%)	
**Urine cytology**			0.102
Normal	21 (34.4%)	76 (47.8%)	
Abnormal	40 (65.6%)	83 (52.2%)	
**eGFR**			0.659
<60	41 (67.2%)	100 (62.9%)	
≥60	20 (32.8%)	59 (37.1%)	
**Adjuvant chemotherapy**			0.960
Yes	29 (47.5%)	78 (49.1%)	
No	32 (52.5%)	81 (50.9%)	
**Ki-67**			0.030
<20%	27 (44.3%)	98 (61.6%)	
≥20%	34 (55.7%)	61 (38.4%)	
**NLR**	2.93 (1.22,14.82)	2.29 (0.67,16.74)	0.004
**PLR**	124.35 (50.06,310.54)	100.8 (35.99 298.60)	0.013
**LMR**	3.58 (1.28,9.79)	3.12 (0.76,8.81)	0.037
**Survival time (months)**	25 (3-136)	45 (2-143)	<0.001

EUR, extraurothelial recurrence; BMI, body mass index; DM, diabetes mellitus; LVI, lymphovascular invasion; NLR, neutrophil-to-lymphocyte ratio; PLR, platelet-to-lymphocyte ratio; LMR, lymphocyte-to-monocyte ratio.

### Predictors of EUR

**Figure 1:**
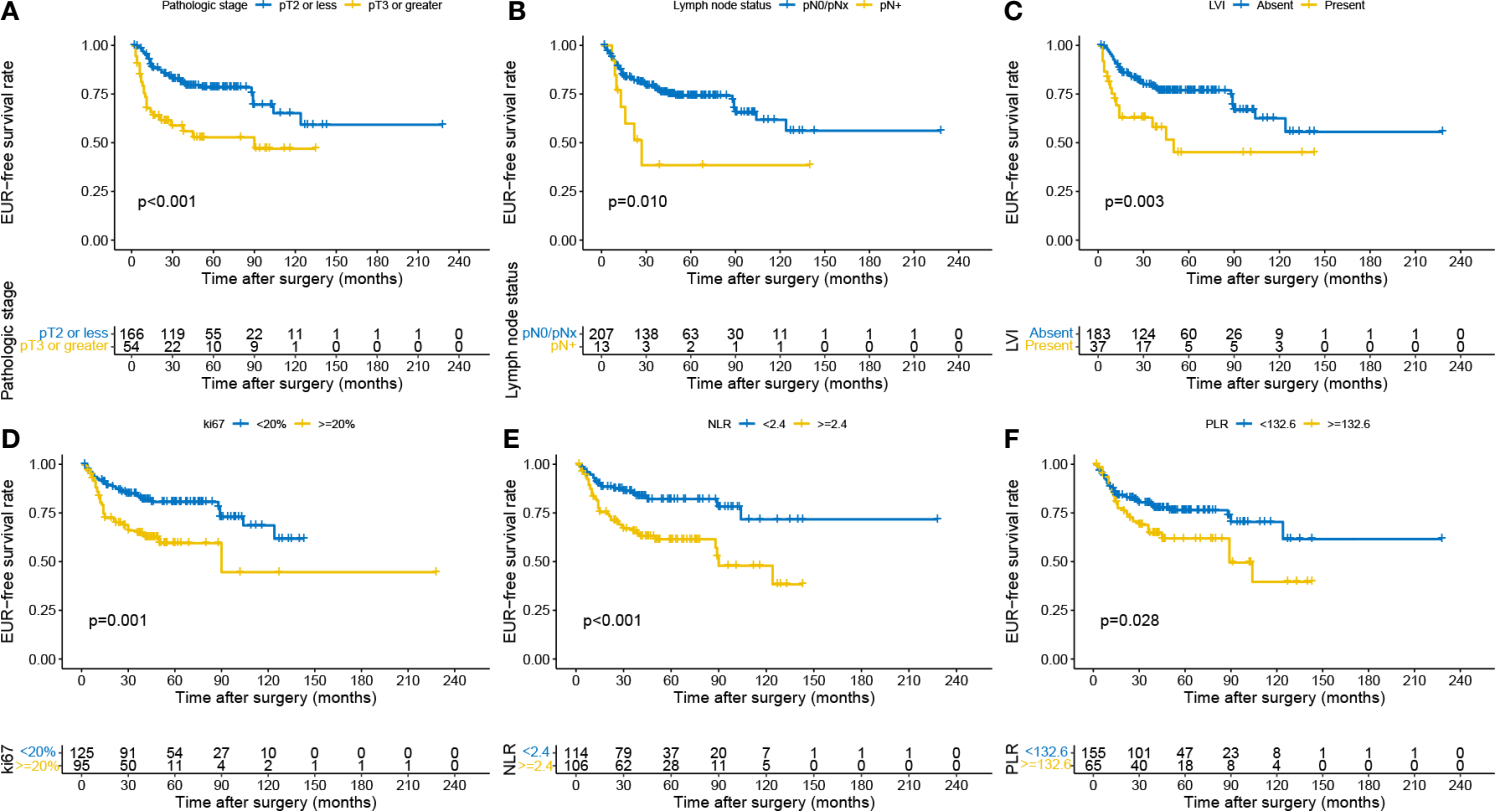
Kaplan–Meier curves for EURFS grouped by risk factors in the patients with UTUC, including pathologic stage **(A)**, lymph node status **(B)**, LVI **(C)**, Ki-67 **(D)**, NLR **(E)**, and PLR **(F)**. EURFS, extraurothelial recurrence-free survival; UTUC, upper urinary tract urothelial carcinoma; LVI, lymphovascular invasion; NLR, neutrophil-to-lymphocyte ratio; PLR, platelet-to-lymphocyte ratio.

Univariate and multivariate Cox regression models were applied to identify the prognostic risk factors related to EUR. The results were presented in **Table 2**. In the univariate Cox analysis, pathologic stage, LN status, tumor grade, hydronephrosis, LVI, urine cytology, Ki-67, NLR, PLR, LMR, and LVI showed a strong association with EUR (*p* < 0.05). After including these relative factors, pathologic stage, LN status, LVI, Ki-67, NLR, and PLR were identified as independent risk factors for EUR based on multivariate Cox analysis. Kaplan–Meier curves were plotted to demonstrate the difference between subgroups divided by these risk factors. The results showed that patients with these risk factors had a shorter EURFS (**Figure 1**).

**Table 2:** Uni- and multivariate analysis of the 220 patients for extraurothelial recurrence-free survival.

Characteristic	Univariate analysis			Multivariate analysis		
	HR	95% CI	*p* value	HR	95% CI	*p* value
**Age (years)**	0.993	0.968-1.019	0.602			
Sex						
Men	Reference					
Women	0.654	0.396-1.082	0.099			
**BMI**	1.003	0.931-1.080	0.944			
History of hypertension						
Yes	Reference					
No	0.948	0.573-1.568	0.836			
History of DM						
Yes	Reference					
No	0.942	0.501-1.773	0.853			
Tumor location						
Ureter	Reference					
Renal pelvis	0.833	0.251-2.765	0.765			
Both	1.068	0.327-3.491	0.913			
Laterality						
Left	Reference					
Right	1.17	0.707-1.934	0.541			
Pathologic stage						
pT2 or less	Reference			Reference		
pT3 or greater	2.658	1.586-4.456	<0.001	2.755	1.554-4.886	<0.001
Lymph node status						
pN0/pNx	Reference			Reference		
pN+	2.694	1.220-5.949	0.014	2.468	1.046-5.822	0.039
Tumor grade						
Low	Reference			Reference		
High	4.145	1.011-16.990	0.048	2.319	0.542-9.931	0.257
Tumor size						
<3cm	Reference					
≥3cm	1.598	0.966-2.641	0.068			
Tumor multifocality						
Absent	Reference					
Present	1.01	0.555-1.838	0.973			
Hydronephrosis						
Absent	Reference			Reference		
Present	1.662	1.004-2.750	0.048	1.416	0.844-2.374	0.188
LVI						
Absent	Reference			Reference		
Present	2.335	1.317-4.141	0.004	1.893	1.038-3.454	0.037
Margin positivity						
Negative	Reference					
Positive	1.972	0.789-4.929	0.146			
Urine cytology						
Normal	Reference			Reference		
Abnormal	1.699	0.999-2.888	0.049	1.505	0.848-2.671	0.163
eGFR						
<60	Reference					
≥60	0.830	0.398-1.712	0.625			
Adjuvant chemotherapy						
Yes	Reference					
No	0.958	0.579-1.586	0.868			
Ki-67						
<20%	Reference			Reference		
≥20%	2.283	1.360-3.832	0.002	2.000	1.160-3.449	0.013
NLR						
<2.4	Reference			Reference		
≥2.4	2.553	1.495-4.361	0.001	2.342	1.333-4.115	0.003
PLR						
<132.6	Reference			Reference		
≥132.6	1.757	1.054-2.928	0.031	2.536	1.437-4.474	0.001
LMR						
<3.2	Reference			Reference		
≥3.2	0.504	0.295-0.864	0.013	0.665	0.376-1.177	0.162

BMI, body mass index; DM, diabetes mellitus; LVI, lymphovascular invasion; eGFR, estimated glomerular filtration rate; NLR, neutrophil-to-lymphocyte ratio; PLR, platelet-to-lymphocyte ratio; LMR, lymphocyte-to-monocyte ratio.

### Survival outcome of patients with EUR

**Figure 2:**
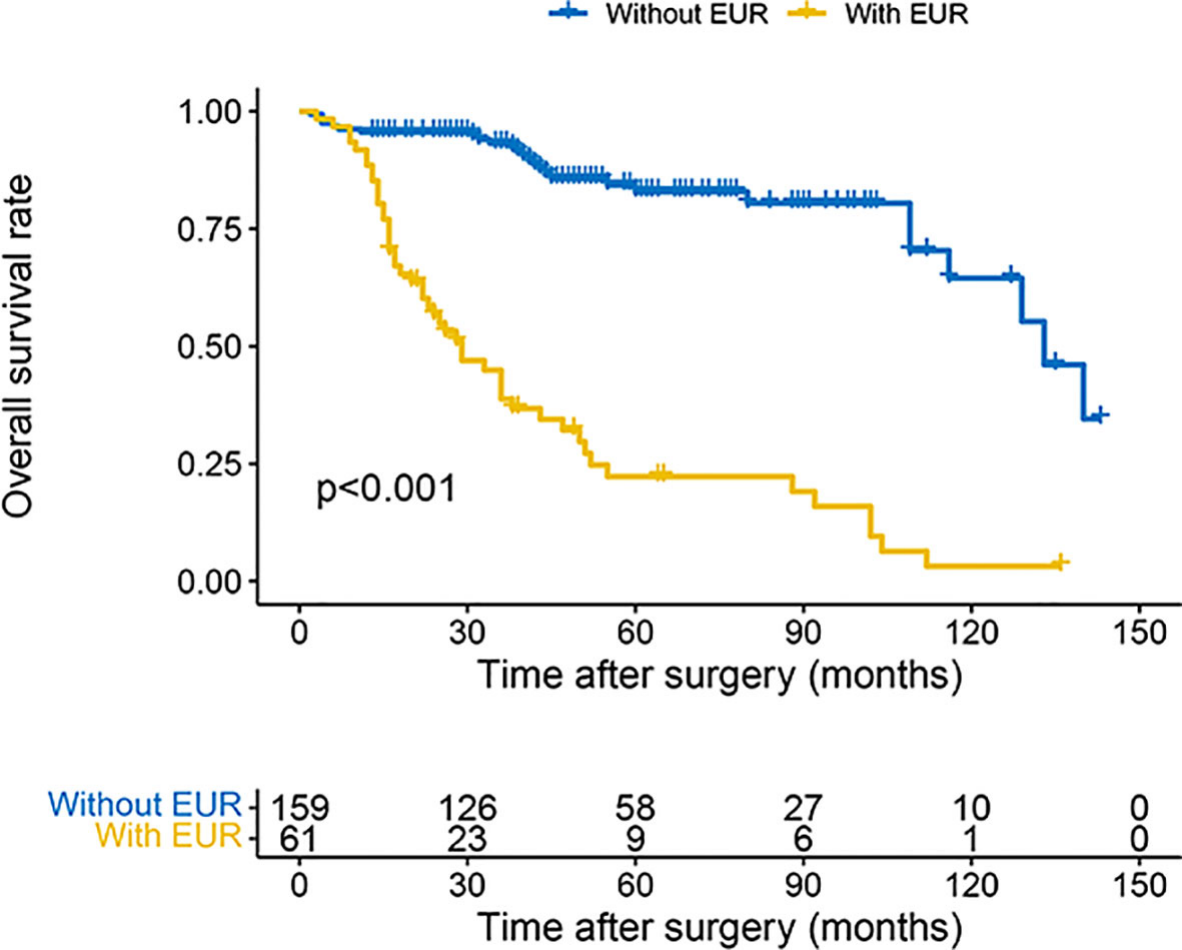
Kaplan–Meier curves for OS of patients with UTUC according to EUR. OS, overall survival; UTUC, upper urinary tract urothelial carcinoma; EUR, extraurothelial recurrence.

**Figure 3:**
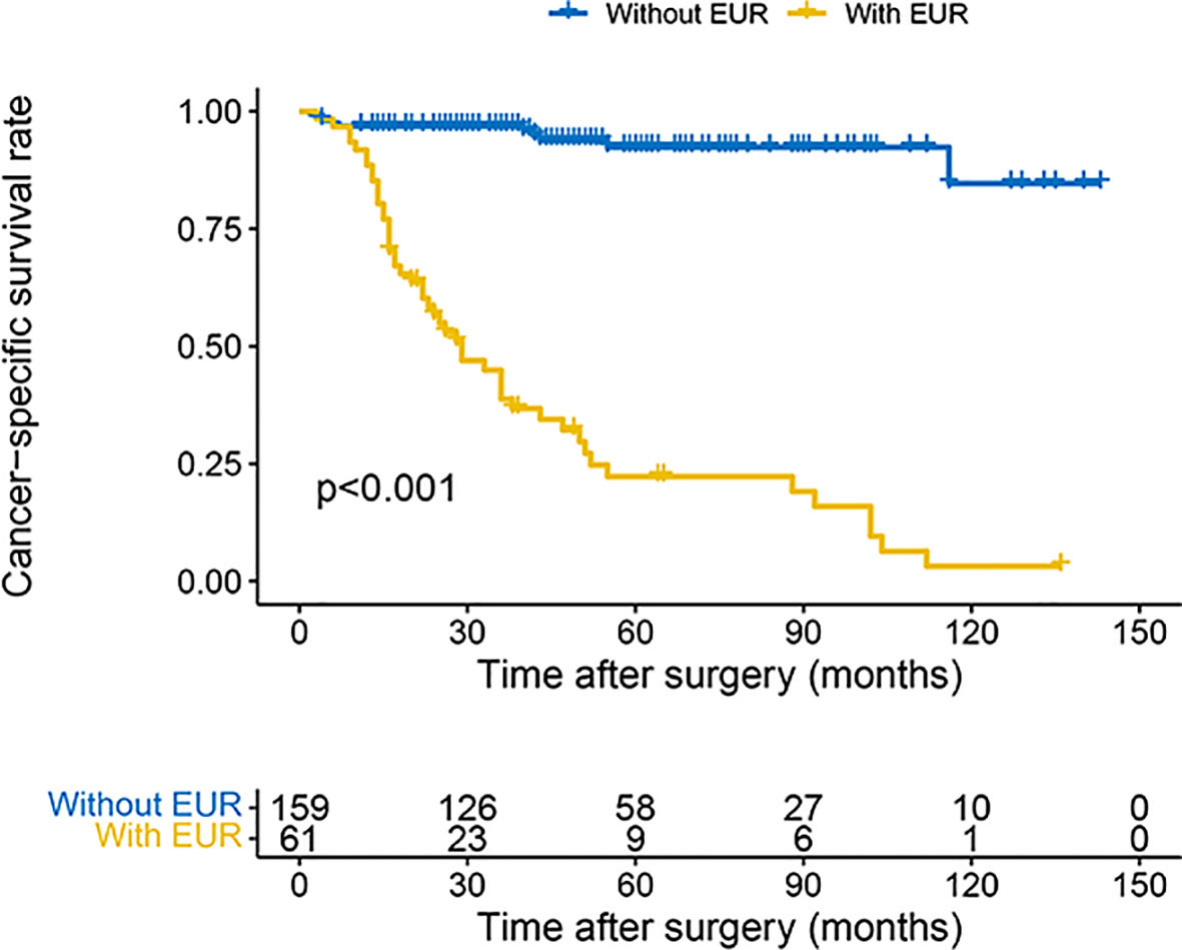
Kaplan–Meier curves for CSS of patients with UTUC according to EUR. CSS, cancer-specific survival; UTUC, upper urinary tract urothelial carcinoma; EUR, extraurothelial recurrence.

The 3- and 5-year OS estimate was 78.0% and 65.2% in all patients, respectively. However, in patients with EUR, 3- and 5-year OS estimate was 38.8% and 22.3%, respectively. Similarly, for CSS, the survival rate for 3 and 5 years was 80.5% and 71.4% in all patients and 38.8% and 22.3% in patients with EUR, respectively. The plots revealed that patients with EUR had significantly worse OS and CSS (**Figures 2**, **3**).

### Risk stratification analysis for EUR

**Figure 4:**
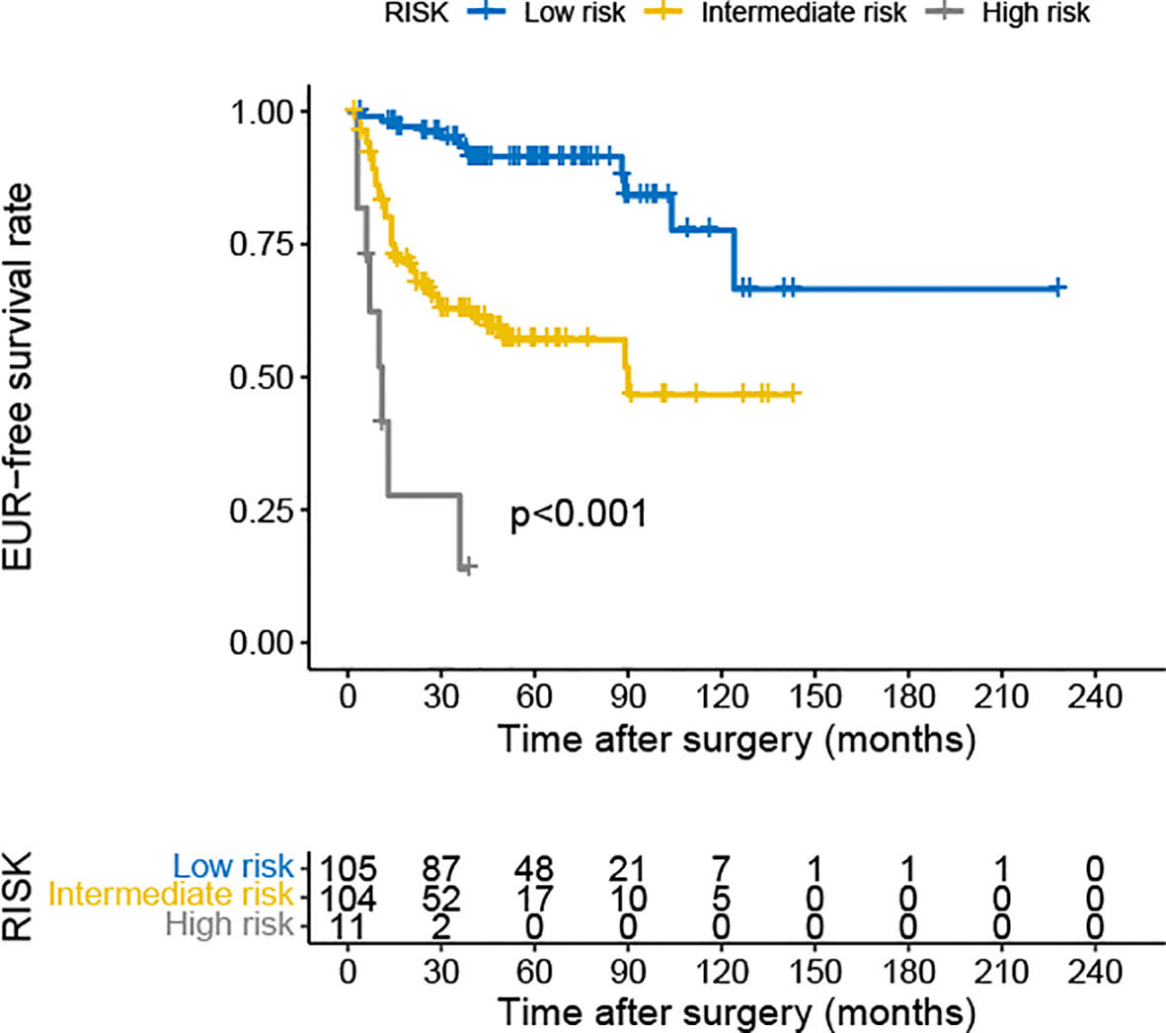
Kaplan–Meier curves for EURFS of patients with UTUC according to risk classification. EURFS, extraurothelial recurrence-free survival; UTUC, upper urinary tract urothelial carcinoma.

The multivariate Cox analysis showed that pT3–4, pN+, LVI, Ki-67 ≥20%, NLR ≥2.4, and PLR ≥132.6 were significant predictors of EUR. Combining the analysis results, we developed a risk classification model according to the number of risk factors (0, 1, or 2 and 3 or more). The low-risk group included patients with 0 or 1 risk factor. Those with two risk factors were included in the intermediate-risk group, and the high-risk group included patients with three or more risk factors. The Kaplan–Meier curves revealed a significant difference in EURFS among the three groups (**Figure 4**). We also performed ROC analysis to evaluate the discriminatory ability of the simple model; the AUC value was 0.769, and sensitivity and specificity were 0.869 and 0.610, respectively (**Supplementary Figure S1**).

## Discussion

Although both UTUC and BC are transitional cell carcinomas in their pathological manifestations, UTUC is more prone to local progression or recurrence after surgery compared to BC (15). Previous studies showed that 20%–30% of patients with UTUC will develop EUR, even if they underwent RNU (16–18). The most common sites of EUR are the lung, bone, and liver (19). However, despite recent advances in adjuvant chemotherapy, which can reduce the risk of EUR to a certain extent, the overall outcome benefit is still unsatisfactory (20). Prognostic models commonly used in clinical practice all cast light on survival outcome. However, patients who experience EUR face a rapid decline in their quality of life and a heavy financial burden. Therefore, accurately screening the high-risk population for postoperative EUR has become the focus and difficulty of clinical attention. Different with other literature reports (18, 21), our results show that pathologic stage, LN status, LVI, Ki-67, NLR, and PLR are independent risk factors.

For the TNM stage of UTUC, the T stage represents the depth of tumor invasion. The higher T stage suggests a higher risk of metastases and worse prognosis. The 5-year CSS rate of patients with pTa/T1 stage is usually over 90%, while that of patients with T2/T3 stage drops to less than 50%, and that of patients with T4 stage is less than 10% (22, 23). In our research, patients with a higher pT stage had a higher incidence of EUR and worse survival outcomes than those with a lower pT stage. Ouzzane et al. (24) reported that the T stage is both a risk factor of local recurrence and distant metastasis in patients with UTUC who underwent RNU. For tumors with a higher T stage, especially those located in the ureter, tumor cells are more likely to penetrate the thinner adventitia of the ureter and invade the vascular lymphatic plexus, making it easier for EUR to occur.

Currently, there is still controversy about the survival benefit and safety of lymph node dissection (LND). Therefore, RNU with concurrent LND is rarely performed in the real world. The guidelines of the European Association of Urology (EAU) recommend that LND should be performed concurrently with surgery for patients with a high risk, but this is based on a low level of evidence (1). Cha et al. (25) reported that the risk of CSS in UTUC patients with LN positivity was approximately 2.23 times higher than that in those with LN negativity. Abe et al. (26) conducted a multicenter study including 293 patients on the relationship between LN status and prognosis. The results showed that for patients with T2 or higher stage, the CSS of patients without LN metastasis (pN0) was higher than that of patients without lymphadenectomy (pNx) (26). Our results also indicate that patients with positive LNs have a worse prognosis and a higher incidence of EUR. In our center, LND was performed in 36 patients with suspected enlarged LNs detected during intraoperative inspection or preoperative images with suspected LN metastasis and only 13 patients had LN metastasis. In view of the above, how to more accurately identify preoperative LN metastases has become a problem that should be solved promptly.

Recent studies have shown that adjuvant chemotherapy could improve oncological outcomes for patients with UTUC (1, 27). Interestingly, our research suggested that adjuvant chemotherapy was not associated with EUR. We reviewed our original data and found that the results could be explained by the following reasons: first, some patients were unable to adhere to the full course of adjuvant chemotherapy due to advanced age combined with underlying diseases or financial reasons, thus affecting the effectiveness of adjuvant chemotherapy; next, some patients were not sensitive to cisplatin-based chemotherapy and develop secondary drug resistance during the course of treatment. Therefore, individualized treatment of UTUC patients after RNU should be the focus of future research.

LVI is currently recognized as an important factor affecting the clinical prognosis of patients with malignant tumors (28). The detection rate of LVI in UTUC is approximately 20% (29, 30). In our study, a total of 37 patients (16.8%) had LVI, which is slightly lower than that reported by Novara et al. (29). We speculate that this discrepancy may be related to the number of tumor tissue samples and the experience of pathologists. Studies have shown that vascular lymphatic invasion is a prerequisite for tumor LN invasion, which can further lead to tumor cells entering the blood and forming micrometastases (31). Margulis et al. (6) and Hurel et al. (32) also confirmed that LVI is an independent predictor of postoperative recurrence and distant metastasis in patients with UTUC, which is in accordance with our result.

Ki-67 is an immunostaining marker of nuclear cell proliferation, which is frequently expressed in several malignancies, such as breast, colon, and ovarian cancers (33, 34). Referring to relevant literature, we defined that Ki-67 overexpression was 20% (33–35). In our study, 43.2% of the patients showed Ki-67 overexpression, which is consistent with the percentage of Ki-67 ≥20% in previous studies (36, 37). The research by Krabbe et al. (33) demonstrated that Ki-67 was a validated independent predictor of recurrence-free survival and CSS in patients with UTUC treated with RNU. Our study also confirmed that Ki-67 overexpression was closely related to EUR. However, due to differences in race, sample size, and laboratory equipment, the results should be interpreted with caution.

The occurrence and development of tumors are known to be associated with the systemic inflammatory response (38, 39). Related cellular components such as neutrophils, lymphocytes, and platelets have been reported to have prognostic value in patients with a variety of cancers (9, 40). In this study, we analyzed some systemic inflammation biomarkers including NLR, PLR, and LMR. Although all of these markers showed significant differences in univariate Cox analysis, only NLR and PLR were identified as independent risk factors of EUR when performing multivariate Cox analysis. Neutrophils are an important component of the inflammatory response and have been found to participate in the suppression of antitumor immune monitoring and extracellular matrix remodeling, promoting the metastasis of cancer cells. Furthermore, elevated neutrophils can produce more inflammatory transmitters, such as interleukin (IL)-1, IL-6, IL-17, vascular endothelial growth factor, and other immunomodulatory transmitters, leading to angiogenesis and the progression of malignant tumors (41). Lymphocytes are also an essential part of the immune response and produce antitumor effects through humoral and cellular immunity. Activated and proliferating lymphocytes secrete interferon-γ (IFN-γ) and tumor necrosis factor-α (TNF-α), which inhibit the proliferation and migration of tumor cells. Therefore, low lymphocyte counts may reflect an impaired host immune function (42). Elevated NLR reflects the body’s inflammatory state and immune function abnormalities, and tumors are more likely to recur and metastasize in patients with this condition. Several studies have reported that preoperative NLR is associated with adverse clinicopathologic features and worse survival outcomes (43, 44). The EAU guidelines have suggested preoperative NLR as a prognostic factor for CSS in UTUC (1).

Platelets are generally considered to be one of the key components in physiological hemostasis. However, studies have confirmed that platelets release pro-inflammatory mediators, such as cytokines and chemokines, which can promote angiogenesis and stimulate the production of stromal growth factors and matrix remodeling enzymes, thereby promoting tumor progression and metastasis (45, 46). Therefore, an increase in PLR indicates that patients have a worse prognosis and the prognostic value of PLR has also been verified in some research (47, 48). NLR and PLR can be conveniently obtained from routine blood tests before surgery. Therefore, these biomarkers could provide potentially prognostic information without increasing costs in clinical application. It is worth mentioning that there is no consensus on the best cutoff values for these inflammation biomarkers. Most research selected the cutoff value of NLR from 2 to 5 and PLR from 120 to 200 (9). The cutoff value in this study was determined by X-tile software and also falls within this range. However, further investigations with large samples are required to define the optimal cutoff value for these inflammation biomarkers.

In order to better stratify patients for risk of EUR, we construct a grading system based on these risk factors. Interestingly, the model can also distinguish the OS and CSS of patients (**Supplementary Figures S2**, **S3**). The application of this classification model will be helpful for developing personalized treatment options and follow-up plans for UTUC patients treated with RNU. For UTUC patients with low-risk EUR, regular bladder instillation may be sufficient instead of adjuvant chemotherapy to avoid overtreatment. However, owing to worse survival outcomes, postoperative follow-up and selection of treatment options need to be more cautious for patients with high-risk EUR. It may be necessary to comprehensively consider multiple treatment options such as chemotherapy, radiotherapy, and immunotherapy to improve the prognosis as much as possible.

The present study has several strengths, as follows: first, the follow-up was relatively long compared to previous studies; in addition, this is the first research concerning the EUR of Chinese patients with UTUC to our knowledge; third, the NLR and PLR were identified as independent risk factors, which can be obtained before surgery compared to other risk factors, thereby guiding further treatment in a timely manner. However, there are several limitations in our study that should be acknowledged. First, selection bias cannot be avoided, since this is a retrospective study with a limited sample size due to the low incidence of UTUC and our strict inclusion criteria. Some variables such as performance status, molecular parameters, and genetic mutations were not collected in our cohort, which require further prospective multicenter trials to focus on. Moreover, the relative standards of blood tests, urine exfoliation cytology, and pathological examinations may have changed over the study period, further affecting the reliability of the collected data. Next, the grading system in our study is simple but does not consider the contribution of each risk factor, in which the coefficient is not applicable in this model. Finally, these results should be interpreted with caution, since the data are from a single center.

## Conclusion

Our study indicates that pathologic stage, LN status, LVI, Ki-67, NLR, and PLR are independent risk factors for EUR after RNU. We have developed a prediction system based on these factors for risk stratification in patients with UTUC, which will assist urologists in making clinical decisions for better survival benefits. Further prospective multicenter trials with extensive follow-up are required to confirm our findings.

## Data availability statement

The original contributions presented in the study are included in the article/**Supplementary Material**. Further inquiries can be directed to the corresponding authors.

## Ethics statement

The studies involving human participants were reviewed and approved by the China-Japan Friendship Hospital. The patients/participants provided their written informed consent to participate in this study.

## Author contributions

(I) study design: ZD, GZ; (II) data collection: CS, HZ, BJ; (III)data analysis and interpretation: YP, YB, ZL; (IV) Article writing: ZL, BJ, YY. All authors contributed to the article and approved the submitted version

## Supplementary material

Supplementary Figure 1 ROC for risk classification in predicting EUR after RNU.

Supplementary Figure 2 Kaplan–Meier curves for OS of patients with UTUC according to risk classification.

Supplementary Figure 3 Kaplan–Meier curves for CSS of patients with UTUC according to risk classification.
