# Supplementary figures and images for: Risk factors for extraurothelial recurrence in upper tract urothelial carcinoma after radical nephroureterectomy: a retrospective study based on a Chinese population
Source: Front Oncol. 2023 Aug 9;13:1164464. doi: 10.3389/fonc.2023.1164464 (PMC10445394; doi:10.3389/fonc.2023.1164464)

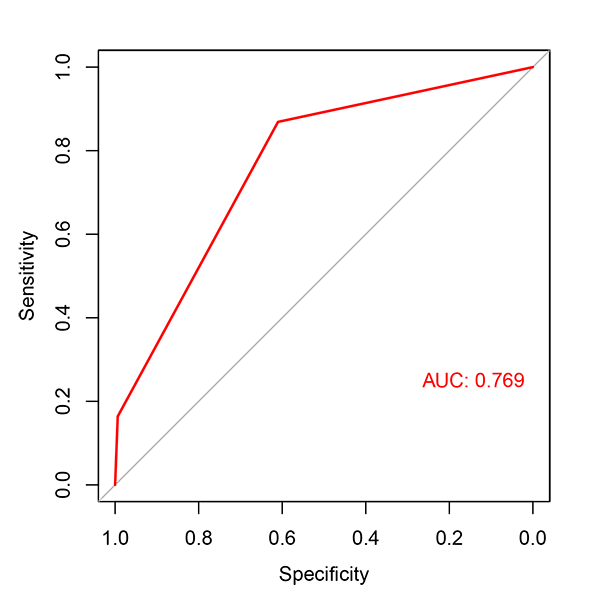

Supplement: Supplementary Figure 1 — ROC for risk classification in predicting EUR after RNU. [file Image_1.tif]

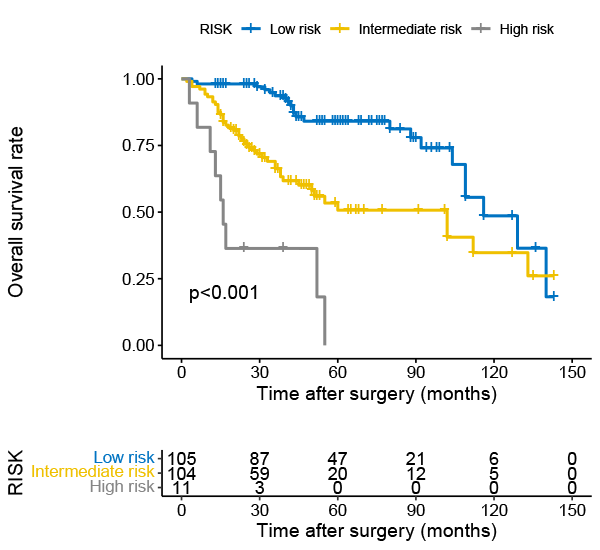

Supplement: Supplementary Figure 2 — Kaplan–Meier curves for OS of patients with UTUC according to risk classification. [file Image_2.tif]

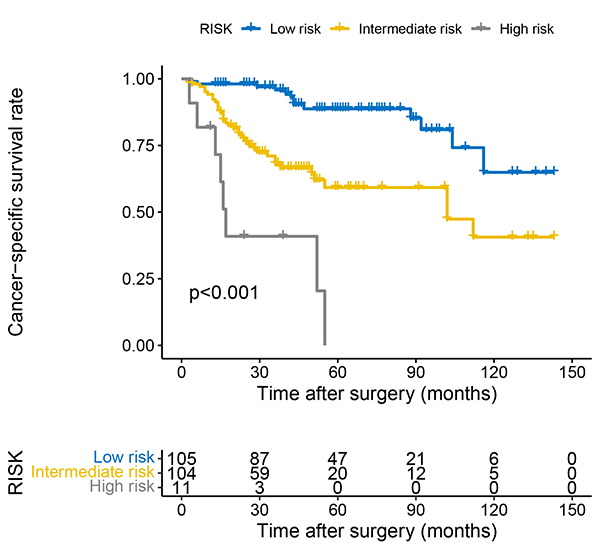

Supplement: Supplementary Figure 3 — Kaplan–Meier curves for CSS of patients with UTUC according to risk classification. [file Image_3.tif]
